# Associations of sleep quality and exercise frequency and the risk of coronary heart disease in Chinese urban elderly: a secondary analysis of cross-sectional data

**DOI:** 10.1186/s12889-023-17077-6

**Published:** 2023-11-08

**Authors:** Jiujing Lin, Huichen Yao, Jia Li, Shoufeng Tian, Xiaoliang Li, Qingzhi Hou

**Affiliations:** 1 School of Public Health and Health Management, Shandong First Medical University and Shandong Academy of Medical Sciences, 6699 Qingdao Rd, Jinan, 250117 Shandong China; 2 Medical Science and Technology Innovation Center, Shandong First Medical University and Shandong Academy of Medical Sciences, Jinan, Shandong China; 3 Cardiology Department, The Third Affiliated Hospital of Shandong First Medical University and Shandong Academy of Medical Sciences, Jinan, Shandong China; 4 Disease Control and Prevention Center of Jinan Shi Zhong District, Jinan, Shandong China

**Keywords:** Initiating sleep, Sleep duration, Sleep disorder, Exercise frequency, Coronary heart disease, Elderly

## Abstract

**Background:**

Sleep quality and exercise frequency are closely associated with coronary heart disease (CHD). Few studies focused on the joint effect of initiating sleep, sleep disorders, and exercise frequency on the risk of CHD in the elderly. We used a secondary data analysis based on Boshan Elderly cross-sectional study. We explored the sleep quality, exercise frequency, and their joint effects on the risk of CHD.

**Methods:**

We collected 678 participants whose age ≥ 60 years old from Boshan District Hospital. We used the Pittsburgh Sleep Quality Index to evaluate the sleep quality and obtained physical examination information from the hospital.

**Results:**

Compared with the non-CHD group, patients with CHD spent more time in initiating sleep (time ≥ 60 min, 34.59% vs*.* 22.93%, *P* = 0.025) and less time exercising (exercise frequency < 1 times/week, 23.90% vs. 17.15%, *P* = 0.024). In multiple logistic regression analysis, sleep latency ≥ 60 min was associated with CHD risk (adjusted OR = 1.83; 95% CI: 1.11, 2.99; *P*-trend = 0.008). The adjusted OR (95% CI) of CHD was 2.24 (1.16, 4.34) for sleep duration < 5 h versus 5–9 h. Compared with exercise frequency < 1 times/week, the adjusted OR for exercise frequency ≥ 1 times/week was 0.46 (95% CI: 0.26, 0.83; *P* = 0.010). In addition, the joint effects of long sleep latency (≥ 60 min) and sleep disorders were associated with CHD (adjusted OR = 3.36; 95% CI: 1.41, 8.02). The joint effect of exercise frequency ≥ 1 times/week and sleep onset latency within normal limits (< 30 min) was also associated with CHD, and the adjusted OR (95% CI) was 0.42 (0.21, 0.87).

**Conclusions:**

Long sleep latency, high frequency of initiating sleep difficulty, sleep disorders, and short sleep duration were positively associated with CHD. In addition, the joint effects of long sleep latency and sleep disorders were positively correlated with CHD incidence. However, the joint effects of exercise frequency ≥ 1 times/week and normal sleep onset latency were negatively associated CHD.

**Supplementary Information:**

The online version contains supplementary material available at 10.1186/s12889-023-17077-6.

## Background

Cardiovascular disease (CVD) is the primary cause of death and disease burden worldwide, especially in China [1]. Coronary heart disease (CHD) is one of the most common disabling and fatal CVDs; prevention of CHD has received great medical attention. Previous studies reported that lifestyles are closely correlated with the risk of CHD [2]. Sleep and exercise are important aspects of lifestyle. In the elderly, lack of exercise and sleep is very common, which can cause physical and psychological problems [3–5].

For the past few years, researchers have increasingly focused on the effects of sleep quality and exercise on metabolic syndrome, diabetes, and CHD [3, 5, 6]. Sleep and exercise have been considered to play an important role in the occurrence and development of CHD. A meta-analysis showed that long sleep duration (> 9 h) is positively correlated with the risk of CHD [7]. Previous studies [3, 8, 9] showed a U-shaped curve for the association of sleep duration with CHD and found that long sleep duration (> 8 h) and short sleep duration (< 4 h) can increase the risk of CHD. In addition, many studies reported that obstructive sleep apnea is a significant risk of CHD [10, 11]. As for the studies about exercise and CHD incidence, Carmen F et al*.* found that regular exercise was positively associated with antiatherogenic effects and could decrease the risk of CHD [4]. Varghese T et al. [12] suggested that 30 min of moderate-intensity exercise 5 days/week is negatively associated with CHD. Other studies found that high-intensity exercise is closely correlated with a low risk of CHD [13]. If the total energy consumption of exercise remains constant, relatively high-intensity exercise has been found to protect the heart better than moderate-intensity exercise [14].

Our study focused on the associations of initiating sleep (sleep latency and frequency of initiating sleep difficulty), sleep disorders, exercise frequency, and their joint effect on the risk of CHD.

## Methods

### Study population

Our cross-sectional study was built in 2020 in Boshan, Shandong, China. We included 1034 participants in our cohort study. All the participants completed baseline characteristics collection and medical examinations at Boshan District Hospital from March 1 to September 16 in 2020. Excluding participants who were missing the complete sleep quality report, and participants with self-reported stroke, cancer, heart failure, cerebrovascular disease or severe medical condition(n = 356), we included 678 participants in the present study. This study was carried out in strict accordance with the Declaration of Helsinki in 1975 and supported by the ethics committee of Shandong First Medical University. The participants carried their ID cards to facilitate registration and follow-up management and signed the informed consent.

### Definition of CHD

CHD was defined as the stenosis of any segment of the left main artery, three subepicardial coronary arteries, and their large branches with diameter > 50% [15]. Coronary angiography is the gold standard in the diagnosis of CHD. We diagnosed CHD by coronary angiography in our study, and data were available through medical records and medical diagnoses.

### Definition of sleep quality and exercise frequency

All participants were assessed with Pittsburgh Sleep Quality Index (PSQI), it contained seven sleep factors, including subjective sleep quality, sleep duration, sleep efficiency, sleep onset latency, daytime dysfunction, sleep disorder, and taking hypnotics, which were used to evaluate the short-term sleep quality (the latter was referred to as sleep quality) [16]. Each sleep factor was scored 0, 1, 2, and 3, the total score ( the sum of seven sleep factors) > 5 distinguished poor sleep quality from good [17] and seven sleep factors were divided into the poor groups when the individual score ≥ 2. Sleep disorder, one of the seven sleep factors, was assessed by the frequency (times/week) of nine self-reported items, including going to the toilet at night, easy to wake up, dyspnea, nightmares, pain and discomfort, cold, heat, easy to wake up, and coughing or snoring during sleep in PSQI [18] and each items was divided into the poor groups when the individual score ≥ 2. Moreover, sleep onset latency, another one of the seven sleep factors, included sleep latency and frequency of initiating sleep difficulty, in our analysis. Sleep latency was divided into ≥ 15 min, 16–30 min, 31–60 min, and ≥ 60 min; frequency of initiating sleep difficulty was divided into 0 times/week, < 1 times/week, 1–2 times/week, > 3times/week, each items was scored 0, 1, 2, and 3, the total score ( the sum of sleep latency.

and frequency of initiating sleep difficulty) > 2 was defined as long sleep onset latency, < 2 was defined as normal limits [18]. Sleep duration included < 5 h, 5–9 h, and > 9 hours [18].

Exercise was defined as keeping moderate-intensity activity more than 30 min per day, such as jogging and walking [4]. Exercise frequency was divided into < 1 times/week, ≥ 1 times/week, and every day, which was evaluated by questionnaires at the same time of physical examination.

### Covariates

Sociodemographic characteristics of participants included age, sex, and education levels; lifestyles included drinking, passive smoking, active smoking (never, former, and current), and exercise frequency (< 1 times/week, ≥ 1 times/week, and every day). The data of lifestyles and other factors were collected by face-to-face structured questionnaires, which were conducted by well-trained workers. Moreover, we focused on the changes in lifestyle of the participants during the epidemic of COVID-19 at home and the non-home isolation period, and the exercise and sleep status data during the non-home isolation period were included in our present study. The questionnaires’ recovery rate reached 100%, which could ensure the authenticity and reliability of the questionnaire’s information and reduced data loss. All of the data input was completed by two workers at the same time.

The medical examinations were performed by professional medical workers. The general health information included body mass index (BMI), resting blood pressure (systolic blood pressure and diastolic blood pressure), hypertension, diabetes, and hyperlipidemia. The physical examinations were directly obtained from the hospital, which prevented the participants from concealing their disease history and ensured the reliability and authenticity of the physical examination information.

Diagnostic criteria of chronic diseases were as follows: (1) hypertension: medically diagnosed hypertension, systolic blood pressure ≥ 140 mmHg or diastolic blood pressure ≥ 90 mmHg three times on different days; (2) diabetes: medically diagnosed diabetes or currently taking antidiabetic medications or fasting blood glucose ≥ 7.0 mmol/L; and (3) hyperlipidemia: medically diagnosed hyperlipidemia or total cholesterol ≥ 6.22 mmol/L or triglycerides > 2.26 mmol/L or high-density lipoprotein (HDL) cholesterol < 1.04 mmol/L or low-density lipoprotein (LDL) cholesterol ≥ 4.14 mmol/L.

### Statistical analyses

Based on the data from this cross-sectional study, we conducted a secondary analysis of the effects of sleep quality, exercise frequency, and their joint effects on the incidence of coronary heart disease. The continuous variables were expressed by means (SD) and percentages for categorical variables in the baseline characteristics between CHD and control. We used the χ^2^ test in categorical variable data and *t*-test in continuous variable data. The logistic regression models were used to analyze the association of different sleep problems with CHD, including unadjusted and adjusted models. The results were expressed by adjusted odds ratio (OR) with 95% confidence interval (CI). The adjusted factors were age, sex, BMI, exercise frequency, hypertension, diabetes, and hyperlipidemia. Each group was adjusted by the other covariates except itself. Linear trend *P*-values were estimated in the adjusted models (*P* < 0.05). We also evaluated the association of sleep latency, frequency of initiating sleep difficulty, sleep duration, and exercise frequency with the risk of CHD in a subgroup analysis by sex. We further explored the joint effects of sleep latency and sleep disorders, as well as initiating sleep and exercise frequency, on the risk of CHD. Taking short sleep latency (< 15 min), non-sleep disorders, good initiating sleep, and exercise frequency < 1 time/week were used as the reference groups. All statistical analyses were performed by IBM SPSS version 25.0. Statistical significance was set at a two-sided *P* < 0.05.

## Results

### Baseline characteristics of participants in the CHD and non-CHD groups

In our study, the average age of included participants was 69.11 ± 8.39 years old. The baseline characteristics of participants including age, BMI, and waist and hipline circumferences significantly differed between the CHD and non-CHD groups (all *P* < 0.05). Compared with age ≤ 68 years, the rate of CHD was higher in the elderly with age > 68 years old (58.49% vs. 41.51%, *P* = 0.035), and the participants with BMI ≥ 24 kg/m^2^ had a higher rate of CHD than those with BMI < 24 kg/m^2^ (67.30% vs. 32.70%, *P* = 0.002). In addition, the waist circumference (89.00 ± 10.94 cm vs. 86.00 ± 10.17 cm) and hipline circumference (100.00 ± 8.00 cm vs. 98.00 ± 7.16 cm) was significantly different between the CHD and non-CHD groups (all *P* < 0.05). Meanwhile, sex and education level were not statistically different between the CHD and non-CHD groups (all *P* > 0.05; Table 1).

**Table 1:** Baseline characteristics of participants with CHD and Non-CHD

Characteristics	CHD	Non-CHD	**χ**^**2**^	*P*
	n (%)	n (%)		
N	159 (23.45)	519 (76.55)		
Age (Years)			4.44	0.035
≤ 68	66 (41.51)	265 (51.06)		
> 68	93 (58.49)	254 (48.94)		
Sex			1.44	0.230
Male	61 (38.36)	227 (43.74)		
Female	98 (61.64)	292(56.26)		
Education level (Years)			3.22	0.200
Illiteracy	17 (10.69)	34 (6.55)		
≤ 5	49 (30.82)	178 (34.30)		
> 5	93 (58.49)	307(59.15)		
BMI (kg/m^2^)^a^			9.35	0.002
< 24	52 (32.70)	241 (46.44)		
≥ 24	107 (67.30)	278 (53.56)		
Waist circumference (cm)[mean (SD)]	89.00 ± 10.94	86.00 ± 10.17		< 0.001
Hipline circumference (cm)[mean (SD)]	100.00 ± 8.00	98.00 ± 7.16		< 0.001

^a^*BMI* Body Mass Index

### Differences in lifestyles and disease history between the CHD and non-CHD groups

Our results showed that exercise frequency (χ^2^ = 7.49, *P* = 0.024), sleep quality (χ^2^ = 6.99, *P* = 0.008), hypertension (χ^2^ = 14.87, *P* < 0.001), and diabetes (χ^2^ = 15.30, *P* < 0.001) were statistically different between the CHD and non-CHD groups. Drinking, passive smoking, active smoking, and hyperlipidemia were not statistically different between the CHD and non-CHD groups (all *P* > 0.05; Table 2).

**Table 2:** The difference of lifestyles and disease history between CHD and Non-CHD

Characteristics	CHD	Non-CHD	**χ**^**2**^	*P*
	n (%)	n (%)		
N	159 (23.45)	519 (76.55)		
Drinking			1.65	0.199
Yes	26 (16.35)	109 (21.00)		
No	133 (83.65)	410 (79.00)		
Passive smoking			1.40	0.236
Yes	26 (16.35)	107 (20.62)		
No	133 (83.65)	412 (79.38)		
Active smoking			5.65	0.059
Never	141 (88.68)	437 (84.20)		
Former	11 (6.92)	28 (5.39)		
Current	7 (4.40)	54 (10.41)		
Exercise frequency			7.49	0.024
< 1 times/week	38 (23.90)	89 (17.15)		
≥ 1 times/week	31 (19.50)	152 (29.29)		
Everyday	90 (56.60)	278 (53.56)		
Sleep quality			6.99	0.008
Good	93 (58.49)	362 (69.75)		
Bad	66 (41.51)	157 (30.25)		
Hypertension			14.87	< 0.001
Yes	91 (57.23)	207 (39.88)		
No	68 (42.77)	312 (60.12)		
Diabetes			15.30	< 0.001
Yes	42 (26.42)	69 (13.29)		
No	117 (73.58)	450 (86.71)		
Hyperlipidemia			2.06	0.152
Yes	100 (62.89)	358 (69.00)		
No	59 (37.11)	161 (31.00)		

### Differences in components of sleep quality between the CHD and non-CHD groups

We also compared the differences of seven sleep factors that determined sleep quality between the CHD and non-CHD groups. The results showed that the proportions of serious sleep disorders (23.67% vs. 13.10%, *P* = 0.004), poor initiating sleep (41.51% vs. 20.09%, *P* = 0.005), and sleep duration < 5 h (11.32% vs. 5.59%, *P* = 0.044) in the CHD group were higher than those in the non-CHD group. By contrast, poor subjective sleep quality (70.44% vs. 79.19%, *P* = 0.024) was lower in the CHD group than in the non-CHD group. The differences in those sleep factors between CHD and non-CHD were statistically significant. However, sleep efficiency (χ^2^ = 2.38, *P* = 0.133), taking hypnotics (χ^2^ = 3.60, *P* = 0.063), and daytime dysfunction (χ^2^ = 0.65, *P* = 0.417) were not significantly different between the CHD and non-CHD groups (all *P* > 0.05; Table 3).

**Table 3:** The analysis of the components of sleep quality with CHD and Non-CHD

Characteristics	CHD	Non-CHD	**χ**^**2**^	*P*
	n (%)	n (%)		
N	159 (23.45)	519 (76.55)		
Subjective sleep quality			5.29	0.024
Good	47 (29.56)	108 (20.81)		
Bad	112 (70.44)	411 (79.19)		
Sleep duration (hours)			6.24	0.044
< 5	18 (11.32)	29 (5.59)		
5–9	133 (83.65)	460 (88.63)		
≥ 9	8 (5.03)	30 (5.78)		
Sleep efficiency			2.38	0.133
Good	115 (72.33)	406 (78.23)		
Bad	44 (27.67)	113 (21.77)		
Sleep disorders			9.62	0.004
None or slight	122 (76.73)	451 (86.90)		
Serious	37 (23.67)	68 (13.10)		
Taking hypnotics			3.60	0.063
Yes	121 (76.10)	430 (82.85)		
No	38 (23.90)	89 (17.15)		
Daytime dysfunction			0.65	0.417
None or light	126 (79.25)	426 (82.08)		
Serious	33 (20.75)	93 (17.92)		
Sleep onset latency			8.62	0.005
Normal	93 (58.49)	368 (70.91)		
Long	66 (41.51)	151 (20.09)		
Sleep latency (minutes)			9.37	0.025
≤ 15	64 (40.25)	258 (49.71)		
16–30	29 (18.24)	110 (21.19)		
31–60	11 (6.92)	32 (6.17)		
≥ 60	55 (34.59)	119 (22.93)		
Initiating sleep difficulty(times/week)			11.34	0.010
0	85 (53.46)	351 (67.63)		
< 1	6 (3.77)	17 (3.27)		
1–2	12 (7.55)	22 (4.24)		
≥ 3	56 (35.22)	129 (24.86)		

### Associations of sleep quality and exercise frequency with the risk of CHD

**Fig. 1:**
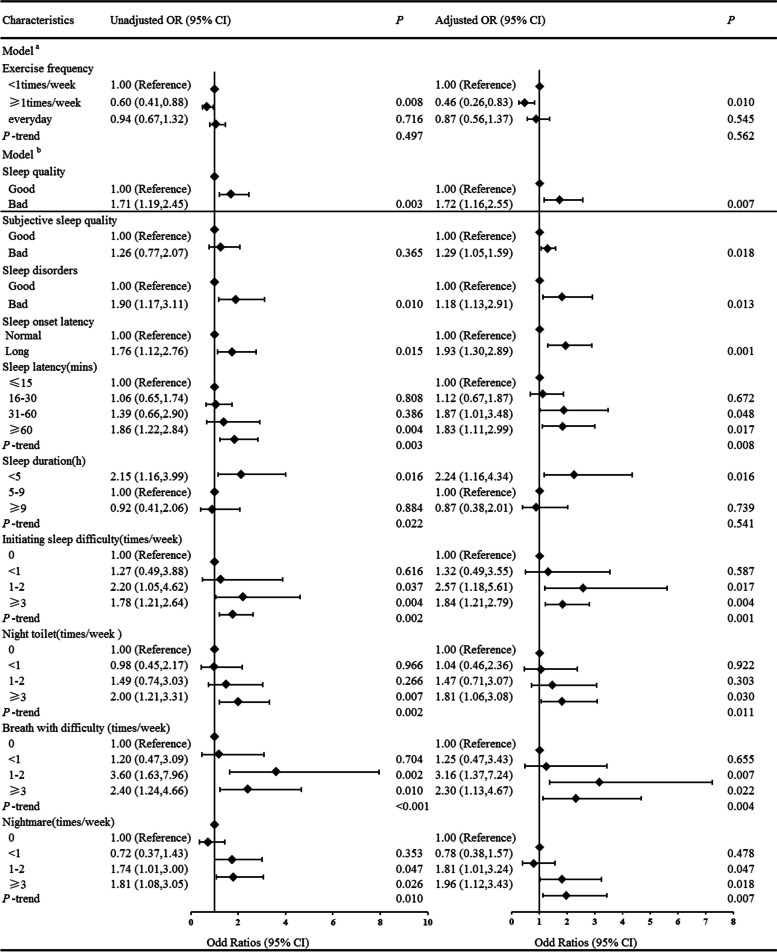
Multivariate logistic regression of lifestyle, disease history and sleep quality and the risk of CHD. Model c: adjusted factors were age, sex, BMI, hyperlipidemia, diabetes and hypertension. Model d: adjusted factors were age, sex, BMI, exercise frequency, hyperlipidemia, diabetes and hypertension. Each group adjusted by the other covariates except itself

We used the logistic regression model to analyze the associations of lifestyle and disease history with CHD incidence, and the results showed that poor sleep quality were positively associated with the risk of CHD (unadjusted OR = 1.71; 95% CI: 1.19, 2.45, adjusted OR = 1.72, 95% CI: 1.16, 2.55; all *P* < 0.05). Exercise frequency ≥ 1 time/week was negatively correlated with CHD (unadjusted OR = 0.60; 95% CI: 0.41, 0.88, adjusted OR = 0.46, 95% CI: 0.26, 0.83, all *P* < 0.05), but *P* of exercise frequency was > 0.05. The confounding factors included age, sex, BMI, exercise frequency, hyperlipidemia, diabetes, and hypertension. Each group was adjusted by the other covariates except itself (Fig. 1).

We examined the associations of seven sleep factors that determined the short sleep quality and CHD incidence. The results showed that sleep disorders (unadjusted OR = 1.90; 95% CI: 1.17, 3.11), poor initiating sleep (unadjusted OR = 1.76; 95% CI: 1.12, 2.76), and sleep duration < 5 h (unadjusted OR = 2.15; 95% CI: 1.16, 3.99) were positively associated with the risk of CHD (all *P* < 0.05). After adjusting for age, sex, BMI, exercise frequency, hyperlipidemia, diabetes and hypertension, sleep disorders (adjusted OR = 1.18; 95% CI: 1.13, 2.91), long sleep onset latency (> 30 min; adjusted OR = 1.93; 95% CI: 1.30, 2.89), and sleep duration < 5 h (adjusted OR = 2.24; 95% CI: 1.16, 4.34) were still statistically related to CHD incidence (Fig. 1). We further examined the associations of the causes of sleep disorders and difficulty initiating sleep with CHD. The results revealed that long sleep latency (≥ 60 min; unadjusted OR = 1.86; 95% CI: 1.22, 2.84; *P* = 0.003, adjusted OR = 1.83; 95% CI: 1.11, 2.99; *P*-trend = 0.008), high frequency of initiating sleep difficulty (≥ 3 times/week; unadjusted OR = 1.78; 95% CI: 1.21, 2.64; *P* = 0.002, adjusted OR = 1.84; 95% CI: 1.21, 2.79, *P*-trend = 0.001), night toilet ≥ 3 times/week (unadjusted OR = 2.00; 95% CI: 1.21, 3.31; *P*-trend = 0.002, adjusted OR = 1.81; 95% CI: 1.06, 3.08; *P*-trend = 0.011), breathing with difficulty at night ≥ 3 times/week (unadjusted OR = 2.40; 95% CI: 1.24, 4.66; *P* < 0.001, adjusted OR = 2.30; 95% CI: 1.13, 4.67; *P*-trend = 0.004), and nightmare ≥ 3 times/week (unadjusted OR = 1.81; 95% CI: 1.08, 3.05; *P*-trend = 0.010, adjusted OR = 1.96; 95% CI: 1.12, 3.43; *P*-trend = 0.007) were positively associated with the risk of CHD (Fig. 1). By contrast, easily waking up, night cough or snore, cold, heat, pain and discomfort, and other factors had no significant correlation with the risk of CHD, and the results are presented in Table S1. The adjusted factors were age, sex, BMI, exercise frequency, hyperlipidemia, diabetes, and hypertension.

### Subgroup analysis by sex of the associations of sleep onset latency, sleep disorders, and exercise frequency with CHD incidence

**Fig. 2:**
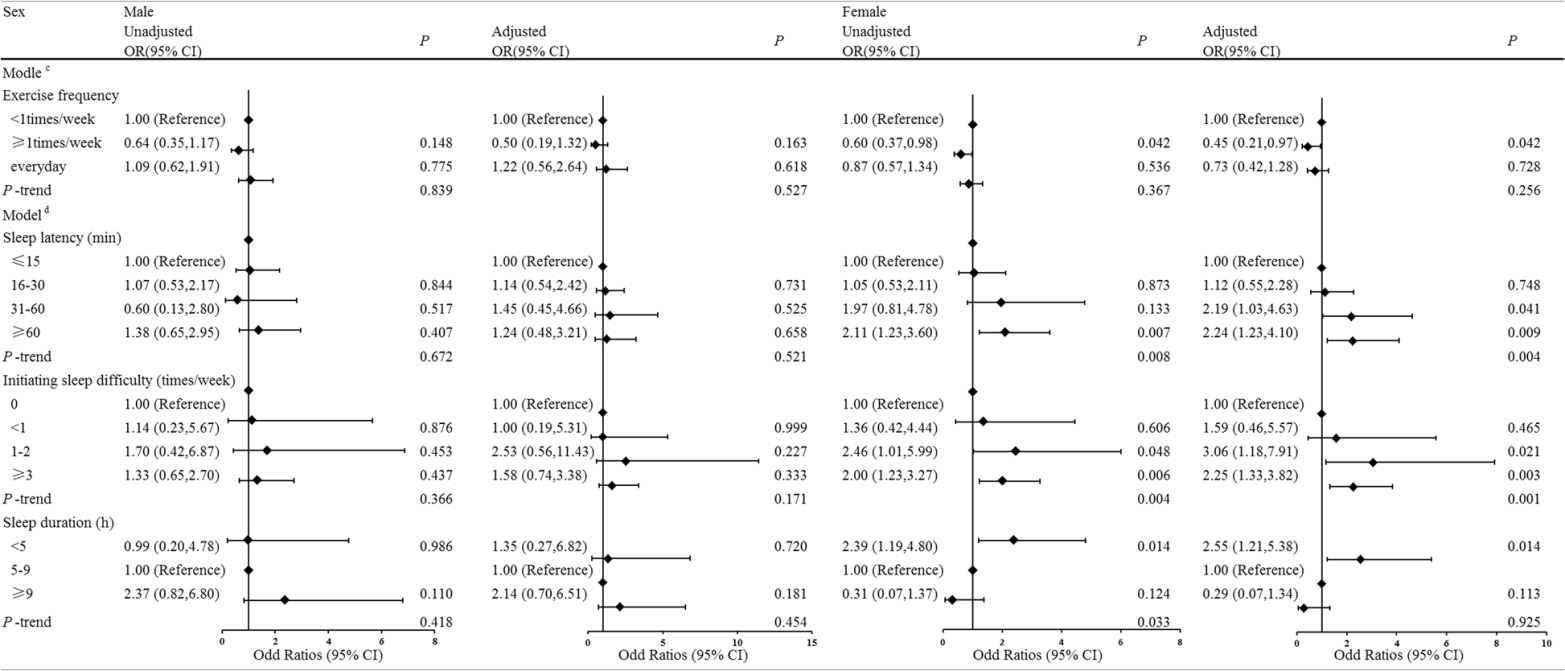
Multivariate regression analysis of initiating sleep and the risk of CHD subgrouping by sex. Model a: adjusted factors were age, BMI, hyperlipidemia, diabetes and hypertension. Model b: adjusted factors were age, BMI, exercise frequency, hyperlipidemia, diabetes and hypertension

In subgroup analysis, after adjusting for age, BMI, exercise frequency, hyperlipidemia, diabetes and hypertension, long sleep latency (≥ 60 min; unadjusted OR = 2.11; 95% CI: 1.23, 3.60; adjusted OR = 2.24; 95% CI: 1.23, 4.10, all *P* < 0.05), short sleep duration (< 5 h; unadjusted OR = 2.39; 95% CI: 1.19, 4.80, adjusted OR = 2.55; 95% CI: 1.21, 5.38, all of *P* < 0.05), and high frequency of initiating sleep difficulty (≥ 3 times/week; unadjusted OR = 2.00; 95% CI: 1.23, 3.27, *P*-trend = 0.004, adjusted OR = 2.25; 95% CI: 1.33, 3.82, *P*-trend = 0.001) were positively associated with the risk of CHD in the female group but not in the male group. Moreover, compared with exercise frequency < 1 time/week, exercise frequency ≥ 1.

time/week (unadjusted OR = 0.60; 95% CI: 0.37, 0.98; adjusted OR = 0.45; 95% CI: 0.21, 0.97, all *P* = 0.042) was negatively associated with the risk of CHD only in the female group. However, after adjusted by age, BMI, hyperlipidemia, diabetes and hypertension, the association had no statistically significant (Fig. 2).

### Joint effects of sleep latency, sleep disorders, sleep onset latency, and exercise frequency on the risk of CHD

**Fig. 3:**
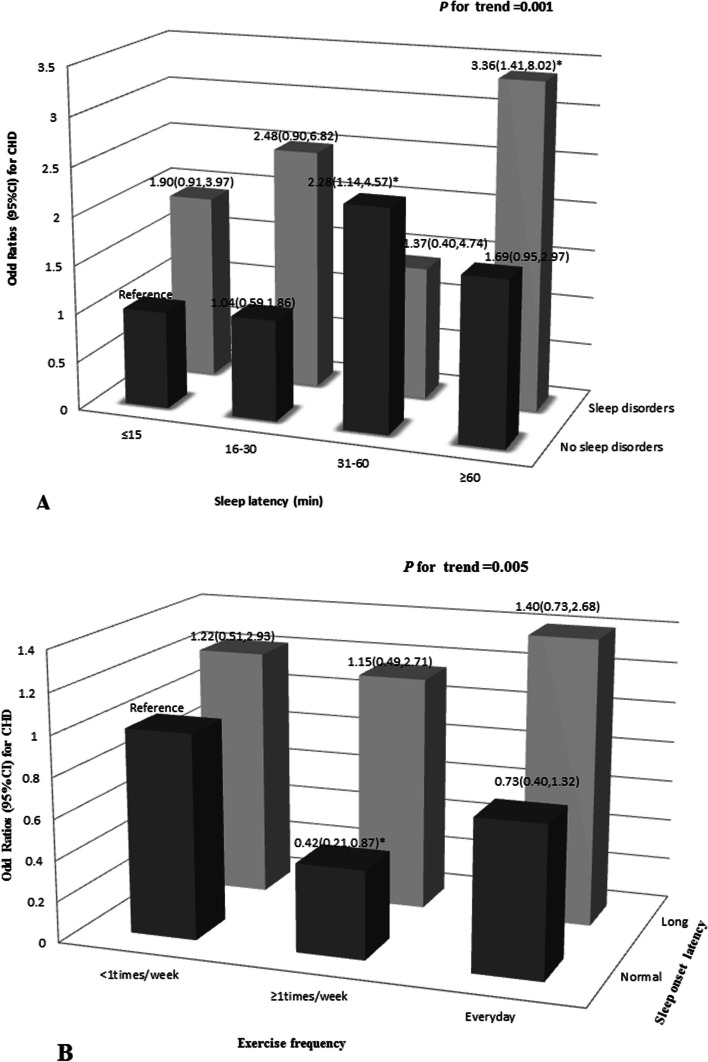
Joint effects of sleep latency and sleep disorders, and exercise frequency and sleep onset latency on the risk of CHD. **A**: The joint effect of (**A**) sleep onset latency (minutes) and sleep disorders and the risk of CHD. Short sleep latency (< 15 min) and non-sleep disorders was reference group. Model A was adjusted by age, sex, BMI, exercise frequency, hyperlipidemia, diabetes and hypertension. **B**: The joint effect of exercise frequency (< 1 times/week, ≥ 1 times/week and Every day) and initiating sleep and the risk of CHD. Good initiating sleep and exercise frequency < 1 times/week was reference group. Model B was adjusted by age, sex, BMI, hyperlipidemia, diabetes and hypertension (*P < 0.05)

We further evaluated the joint effects of sleep latency (minutes) and sleep disorders (yes or no) on the risk of CHD. Compared with short sleep latency (≤ 15 min) and no sleep disorders, participants who reported both long sleep latency (≥ 60 min) and sleep disorders (OR = 3.36, 95% CI: 1.41, 8.02) and both sleep latency 31–60 min and no sleep disorders (OR = 2.28, 95% CI: 1.14, 4.57) were positively associated with CHD incidence. The adjusted factors were age, sex, exercise frequency, hyperlipidemia, diabetes, and hypertension (Fig. 3A). We found that the joint effect of exercise frequency ≥ 1 time/week and sleep onset latency within normal limits (< 30 min) was negatively associated with the risk of CHD (OR = 0.42, 95% CI: 0.21, 0.87). This model was adjusted for age, sex, hyperlipidemia, diabetes, and hypertension (Fig. 3B).

## Discussion

We used a secondary data analysis based on Boshan Elderly cross-sectional study to explore the relationship between sleep quality and exercise frequency and their joint effect on CHD incidence. And we focused on the changes in lifestyle of the participants during the epidemic of COVID-19 at home and the non-home isolation period, and the exercise and sleep status data during the non-home isolation period were included in our present study and explored the relationship between sleep quality and exercise frequency and their joint effect on CHD incidence. Our study revealed that long sleep latency (≥ 60 min), high frequency of difficulty initiating sleep (≥ 3 times/week), sleep disorders, and short sleep duration (< 5 h/night) were positively associated with the risk of CHD. By contrast, exercise frequency ≥ 1 times/week was negatively associated with CHD incidence. In addition, we found that the joint effect of long sleep latency and sleep disorders was positively correlated with the risk of CHD. Moreover, the joint effect of exercise frequency ≥ 1 times/week and good initiating sleep was negatively associated with the risk of CHD.

In our cross-sectional study, we evaluated the sleep quality of participants by PSQI. In most studies, the overall sleep quality was evaluated by the total score of PSQI, which is a one-dimensional model. Previous studies showed that PSQI could provide a multidimensional model to measure sleep quality [19]. In the present study, we not only used the total score of PSQI to evaluate the overall sleep quality but also used the multidimensional model to evaluate the subjective sleep quality, sleep duration, sleep efficiency, initiating sleep, daytime dysfunction, sleep disorders, and taking hypnotics.

Sleep quality plays an important role in regulating metabolism, immunity, cardiovascular activity, and other functions, especially in the elderly [20, 21]. Fukuoka R et al. [22] found that sleep deprivation was significantly associated with the risk of CHD, and the mechanism was explored by regulating the pro-inflammatory process and the secretion of pro-inflammatory cytokines. The concentrations of serum cytokines such as tumor necrosis factor-α, interleukin (IL-1, IL-6, and IL-17), and C-reactive protein were higher in patients with sleep disorders than in patients with good sleep, which could increase the hypothalamus-hypothesis-adrenal axis stress response, elevate blood pressure, and lead to chronic inflammation [20, 23, 24], all of which could increase the risk of CHD. Previous studies reported that sleep disorders have adverse effects on metabolism and endocrine function [25]. Apnea during sleep can decrease blood oxygen saturation and lead to dyspnea, which can trigger cardiovascular events. In addition, sleep apnea has been identified as a risk factor of CHD [26, 27], which was in accordance with our present study.

Moreover, long-term initiating sleep difficulty and long sleep latency can increase the risk of depression. Jaussent et al*.* found that initiating sleep difficulty lasting four years was a predictor for depression in the elderly [28]. Previous studies reported that depression was one of the risk factors of CHD [29, 30]. Moreover, Ariyo et al*.* also found that depression was a significant risk factor of CHD in the elderly [31]. Biologically, initiating sleep difficulty causes negative effects on neural activity [21, 32] and changes in circadian rhythm [33]. Recent studies reported that circadian rhythm disorder increases the risk of CHD and other CVDs [34, 35]. Our study not only revealed that long sleep latency and sleep disorders were independent risks of CHD but also further revealed that their joint effect had stronger effects on the risk of CHD than their individual effects. In our subgroup analysis, we found that initiating sleep difficulty was the risk factor of CHD in the female group but not in the male group. Sands-Lincoln et al*.* found that women with poor sleep quality had a higher risk of CHD than women with normal sleep quality [36]. Previous studies compared the sleep status at different stages of menopause in women and found that sleep disorder was more common in postmenopausal females than in premenopausal females [37–39], and this result was in accordance with the findings of our present study.

As for exercise frequency and CHD incidence, Myers J et al*.* found that exercise plays a negative independent effect on the risk of cardiovascular events. It was also able to modify other risk factors for CHD. With regular exercise, LDL cholesterol levels and non-HDL have been revealed to decrease by 3.0–6.0 and 6 mg/dL, respectively [40]. Yusuf S e*t al.* also found similar results, the OR (95% CI) of CHD was 0.86 (0.76, 0.97) for physical activity [41]. Our present study found that exercise frequency ≥ 1 times/week was an independent protective factor of CHD, which was in accordance with Wisløff’s study [42]. Interestingly, we also found that the joint protective effect of exercise frequency ≥ 1 times/week and good initiating sleep was stronger than their independent effects.

Interestingly, our study also revealed that short sleep duration could increase the risk of CHD. In logistic regression analysis, after adjusting for age, sex, exercise frequency, hypertension, diabetes, and hyperlipidemia, we found that short sleep duration was an independent risk for CHD. In subgroup analysis, similar results were found for women but not for men. Previous research showed a U-shaped curve for associations of sleep duration with CHD [43]; in the sleep duration of 7–8 h, the OR for the risk of CHD was the lowest. In the Dongfeng Tongji cohort study, compared with 7–8 h of sleep, long sleep duration significantly increased the risk of CHD in men but not in women [9, 44]. Amagai Y and Hoevenaar-Blom MP found that a short duration (< 6 h) is a risk factor of CHD, but it was not significant in long sleep duration, which was in accordance with our study [45, 46]. Meanwhile, some studies have shown that short sleep duration could increase the risk of CHD; subgroup analysis by sex revealed that the difference was still statistically significant in the female group but not in the male group [9]. Hormone secretion levels demonstrated sex differences during sleep and different responses to stress [47], which could lead to sex differences in CHD incidence.

The strengths of our investigation were as follows. First, we explored the associations between sleep latency and the risk of CHD in the elderly. Second, our study explored the joint effects of sleep latency and sleep disorders, exercise frequency, and initiating sleep on the risk of CHD. Finally, we evaluated sleep quality and CHD incidence in multiple dimensions with PSQI. However, our study had some limitations. First, this study was a secondary data analysis based on cross-sectional study, which provided insufficient capacity for lasting effects on CHD incidence and causality cannot be inferred, and the possible bidirectional relationships sleep and exercise may have with CHD risk. Second, we only collected data on exercise frequency, and information containing exercise intensity was lacking. Third, although some confounders were adjusted in the main analysis process, the confounding effect of other risk factors, such as the environmental factors of participants, could not be completely excluded, and the causal relationship could not be determined. The last, this study was the lack of assessment of anxiety and depression, sleep apnea which is significant factors common in elderly patients with CHD interfering with sleep quality and the interference of these factors could not be excluded. Therefore, in the future, we will follow up the participants and conduct a longitudinal study that can combine various biochemical indexes and laboratory research results of the participants to further draw an exact conclusion.

## Conclusions

Difficulty initiating sleep, sleep disorders, and exercise frequency were found to be associated with the risk of CHD. Long sleep latency, high frequency of difficulty initiating sleep, short sleep duration, and sleep disorders were independently associated with CHD. The joint effects of exercise frequency ≥ 1 times/week and good initiating sleep were negatively associated with CHD, and the joint effects of long sleep latency (≥ 60 min) and sleep disorders were positively associated with CHD incidence. Our study highlighted that sleep latency, good sleep quality, and exercise frequency had important effects on CHD prevention, especially in the elderly, which could have significant effects on public health. However, our results were not absolute, and it was still necessary to continue to follow up the participants and conduct longitudinal studies to further draw firm conclusions.

### Supplementary Information

**Additional file 1:** **Supplemental Table 1.** The differences of the components of sleep quality with CHD and Non-CHD.

## Data Availability

The datasets used and/or analysed during this study are available from the corresponding author on reasonable request.

## Abbreviations

BSECS: Boshan Elderly Cohort Study
PSQI: Pittsburgh Sleep Quality Index
CHD: Cardiovascular disease
OSAS: Obstructive sleep apnea
BMI: Body mass index
CI: Confidence interval
OR: Odds ratio
SD: Standard deviation
LDL: Low-density lipoprotein
HDL: High- density lipoprotein
