# Supplementary material for: Associations of sleep quality and exercise frequency and the risk of coronary heart disease in Chinese urban elderly: a secondary analysis of cross-sectional data
Source: BMC Public Health. 2023 Nov 8;23:2199. doi: 10.1186/s12889-023-17077-6 (PMC10631020; doi:10.1186/s12889-023-17077-6)
Supplement: Supplementary file 1 — Additional file 1: Supplemental Table 1. The differences of the components of sleep quality with CHD and Non-CHD. [file 12889_2023_17077_MOESM1_ESM.docx]

Supplemental Table 1

The differences of the components of sleep quality with CHD and Non-CHD

| Characteristics | Unadjusted OR (95% CI) | *P* | Adjusted OR (95% CI) | *P* |
| --- | --- | --- | --- | --- |
| Initiating sleep difficulty(times/week) | |  |  |  |
| 0 | 1.00 (Reference) |  | 1.00 (Reference) |  |
| <1 | 1.27 (0.49,3.88) | 0.616 | 1.32 (0.49,3.55) | 0.587 |
| 1-2 | 2.20 (1.05,4.62) | 0.037 | 2.57 (1.18,5.61) | 0.017 |
| ≥3 | 1.78 (1.21,2.64) | 0.004 | 1.84 (1.21,2.79) | 0.004 |
| *P*-trend |  | 0.002 |  | 0.001 |
| Night toilet (times/week ) | |  |  |  |
| 0 | 1.00 (Reference) |  | 1.00 (Reference) |  |
| <1 | 0.98 (0.45,2.17) | 0.966 | 1.04 (0.46,2.36) | 0.922 |
| 1-2 | 1.49 (0.74,3.03) | 0.266 | 1.47 (0.71,3.07) | 0.303 |
| ≥3 | 2.00 (1.21,3.31) | 0.007 | 1.81 (1.06,3.08) | 0.030 |
| *P*-trend |  | 0.002 |  | 0.011 |
| Breath with difficulty (times/week) | |  |  |  |
| 0 | 1.00 (Reference) |  | 1.00 (Reference) |  |
| <1 | 1.20 (0.47,3.09) | 0.704 | 1.25 (0.47,3.43) | 0.655 |
| 1-2 | 3.60 (1.63,7.96) | 0.002 | 3.16 (1.37,7.24) | 0.007 |
| ≥3 | 2.40 (1.24,4.66) | 0.010 | 2.30 (1.13,4.67) | 0.022 |
| *P*-trend |  | <0.001 |  | 0.004 |
| Nightmare(times/week) | |  |  |  |
| 0 | 1.00 (Reference) |  | 1.00 (Reference) |  |
| <1 | 0.72 (0.37,1.43) | 0.353 | 0.78 (0.38,1.57) | 0.478 |
| 1-2 | 1.74 (1.01,3.00) | 0.047 | 1.81 (1.01,3.24) | 0.047 |
| ≥3 | 1.81 (1.08,3.05) | 0.026 | 1.96 (1.12,3.43) | 0.018 |
| *P*-trend |  | 0.010 |  | 0.007 |
| Pain and discomfort(times/week) | |  |  |  |
| 0 | 1.00 (Reference) |  | 1.00 (Reference) |  |
| <1 | 0.87 (0.37,2.03) | 0.739 | 0.81 (0.34,1.97) | 0.648 |
| 1-2 | 1.74 (1.01,3.00) | 0.082 | 1.64 (0.65,4.13) | 0.292 |
| ≥3 | 1.81 (1.08,3.05) | 0.109 | 1.45 (0.67,3.67) | 0.438 |
| P-trend |  | 0.001 |  | 0.310 |
| Cold (times/week) |  |  |  |  |
| 0 | 1.00 (Reference) |  | 1.00 (Reference) |  |
| <1 | 0.53 (0.23,1.20) | 0.126 | 0.53 (0.23,1.22) | 0.136 |
| 1-2 | 0.61 (0.17,2.11) | 0.431 | 0.69 (0.19,2.48) | 0.565 |
| ≥3 | 2.53 (1.33,4.83) | 0.005 | 2.42 (1.20,4.89) | 0.014 |
| P-trend |  | 0.064 |  | 0.105 |
| Easily wake up (times/week) | |  |  |  |
| 0 | 1.00 (Reference) |  | 1.00 (Reference) |  |
| <1 | 0.63 (0.30,1.35) | 0.237 | 0.67 (0.31,1.47) | 0.323 |
| 1-2 | 1.32 (0.76,2.30) | 0.327 | 1.31 (0.73,2.35) | 0.362 |
| ≥3 | 1.49 (1.00,2.21) | 0.050 | 1.41 (0.92,2.16) | 0.111 |
| P-trend |  | 0.002 |  | 0.079 |
| Night cough or snore (times/week) | |  |  |  |
| 0 | 1.00 (Reference) |  | 1.00 (Reference) |  |
| <1 | 1.51 (0.75,3.07) | 0.250 | 161 (0.76,3.42) | 0.215 |
| 1-2 | 1.26 (0.61,2.58) | 0.532 | 0.99 (0.45,2.21) | 0.985 |
| ≥3 | 1.67 (1.04,2.68) | 0.035 | 1.56 (0.93,2.61) | 0.095 |
| P-trend |  | 0.064 |  | 0.113 |
| Hot (times/week) |  |  |  |  |
| 0 | 1.00 (Reference) |  | 1.00 (Reference) |  |
| <1 | 0.54 (0.26,1.13) | 0.101 | 0.56 (0.26,1.18) | 0.126 |
| 1-2 | 1.06 (0.52,2.16) | 0.871 | 1.05 (0.50,2.22) | 0.896 |
| ≥3 | 1.96 (1.02,3.76) | 0.044 | 1.79 (0.79,3.24) | 0.190 |
| P-trend |  | 0.010 |  | 0.441 |
|  |  |  |  |  |
